# Influence of sarcopenia on postoperative complications and long-term survival in pancreatic cancer patients undergone pancreaticoduodenectomy

**DOI:** 10.3389/fnut.2024.1434630

**Published:** 2024-07-04

**Authors:** Guangzhen Qu, Chuanguo Zhou, Yong Zhang, Shao-Cheng Lyu, Ren Lang

**Affiliations:** ^1^Department of Interventional Radiology, Beijing Chao-Yang Hospital Affiliated with Capital Medical University, Beijing, China; ^2^Department of Hepatobiliary and Pancreaticosplenic Surgery, Beijing Chao-yang Hospital Affiliated with Capital Medical University, Beijing, China

**Keywords:** sarcopenia, skeletal muscle index, pancreatic cancer, pancreaticoduodenectomy, complications

## Abstract

**Background:**

Sarcopenia has the potential to impact the postoperative results and extended prognosis of various types of tumors. Nevertheless, the specific impact of sarcopenia on the postoperative results and long-term survival of pancreatic cancer (PC) following pancreaticoduodenectomy (PD) remains inadequately elucidated. This study investigates the significance of sarcopenia according to various Asian standards on postoperative complications and long-term prognosis in PC patients who have undergone PD.

**Methods:**

This retrospective study systematically analyzed patients with PC who underwent PD from January 2015 to December 2022. Sarcopenia was diagnosed by the skeletal muscle index (SMI) obtained by the skeletal muscle area normalized for height squared on the third lumbar vertebra on computed tomography (CT) images. Univariate and multivariate logistic regression analysis were performed to analyze the correlation between sarcopenia and postoperative complications, while Cox regression analysis was utilized to explore the influence of sarcopenia on overall survival (OS) and recurrence-free survival (RFS) in PC patients after PD.

**Results:**

We enrolled 162 patients with PC after PD (92 males and 70 females, mean age: 63.78 ± 10.27 years), including 83 and 79 patients with sarcopenia and non-sarcopenia, respectively. Compared with non-sarcopenia patients, sarcopenia exhibited higher rates of recurrence rate (75% versus 59%, *p* = 0.039). Univariate and multivariate logistic regression analysis showed that sarcopenia did not affect the incidence of complications in patients with PC after PD in three Asian sarcopenia criteria. Multivariate Cox regression analysis indicated that sarcopenia was an independent risk factor for OS (hazard ratio [HR]: 2.49, 95% confidence interval [CI]: 1.73–3.60, *p* < 0.001) and RFS(hazard ratio [HR]: 1.70, 95%confidence interval [CI]: 1.12–2.50, *p* = 0.012) of PC patients with PD in Japanese Society of Hepatology criteria. Meanwhile, according to the Asian pancreatic cancer population standard, sarcopenia is an independent risk factor affecting the long-term OS (hazard ratio [HR]: 2.59, 95% confidence interval [CI]: 1.80–3.70, *p* < 0.001) and RFS (hazard ratio [HR]: 2.00, 95% confidence interval [CI]: 1.36–3.00, *p* < 0.001) of PC after PD. While sarcopenia is recognized as a risk factor for OS (hazard ratio [HR]: 1.81, 95% confidence interval [CI]: 1.08–3.10, *p* = 0.025) in PC patients based on the Fujiwara criteria, it is not found to be associated with RFS (hazard ratio [HR]: 1.60, 95% confidence interval [CI]: 0.90–3.00, *p* = 0.10). The model based on sarcopenia and clinical characteristics has high predictive ability for OS and RFS.

**Conclusion:**

Various Asian diagnostic criteria do not link sarcopenia with postoperative complications in PC patients after PD. Nevertheless, sarcopenia remains a significant independent risk factor for long-term survival, and its combination with clinical characteristics can aid clinicians in predicting long-term survival outcomes.

## Introduction

Pancreatic cancer (PC), a highly fatal malignancy, is becoming the second leading cause of cancer death in the next few decades. The 5-year survival rate is less than 10%, and nearly 80% of the patients have been unable to be removed or have distant metastasis at the time of diagnosis (1). In the past decade, the improvement of the accuracy of diagnostic approaches, the individualization of perioperative management, the progress of radiotherapy technology and systemic treatment of advanced diseases have made relevant but only modest progress in patient outcomes. Although the progress of surgical techniques and adjuvant chemoradiotherapy strategies has made appropriate progress in improving resectability and survival outcomes, the long-term survival of PC patients is still unsatisfied (2). The increasing incidence rate and the younger population of patients pose a serious challenge to the comprehensive and individualized treatment of PC and to improve the outcomes. For the long-term prognosis of PC patients after pancreatoduodenectomy (PD), many studies focus on the clinicopathological characteristics, including tumor differentiation, vascular invasion, lymph node metastasis and tumor size (3, 4). However, to predict the prognosis of patients with PC after PD, the predictive ability of the above clinicopathological characteristics can no longer be further improved, and it is slightly insufficient to consider only the clinicopathological characteristics.

The outcomes of patients with PC not only depend on the clinicopathological characteristics and treatment, but also are closely related to the nutrition and performance status. Sarcopenia, characterized by decreased skeletal muscle index (SMI), descend muscle strength and poor physical performance, has become an important indicator of poor prognosis in cancer patients. It affects the adverse reactions of chemotherapy, poor performance status and tumor progression (5). It is convenient to analyze the skeletal muscle mass at the level of the third lumbar vertebra based on the skeletal muscle index data obtained by computer tomography (CT), and it is widely used in the prognosis analysis of a variety of tumors (6–8). In malignancy, the prevalence of sarcopenia varies among different types of tumors and disease stages. The incidence of sarcopenia was also different in cancer patients who underwent curative surgery. The incidence of sarcopenia was 75/471 (16%) in breast cancer, 75/186 (40.3%) in hepatocellular carcinoma, and 85/256 (33%) in cholangiocarcinoma (9–11). A meta-analysis by Zalite et al. (12) showed that in normal weight and obese patients with pancreatic cancer, the distribution range of sarcopenia was 29.7–65% and 16.2–67%, respectively. The reasons may be high activation of host inflammatory response, disorder of metabolic activity, lack of endocrine secretion and exocrine trypsin (13). At present, the results of some studies on the effect of sarcopenia on the complications and long-term prognosis of PC after PD are inconsistent. Pecorelli (14) deems that sarcopenia will not affect the complications of PC after PD, while Nishida’s study (15) believes that sarcopenia is an independent risk factor for clinical related pancreatic fistula after PC surgery. In the meta-analysis of 43 studies included by Bundred et al. (16), 10 studies reported the impact of preoperative sarcopenia on the postoperative results of PC. Sarcopenia was associated with poor overall survival (hazard ratio: 1.95; 95% confidence interval, 1.54–2.05), but not with the incidence of postoperative complications (odds ratio: 0.96; 95% confidence interval, 0.78–1.19). Furthermore, most of these studies are aimed at the western population, and few studies are based on the Asian population.

Our study explored the impact of sarcopenia in different Asian criteria on the postoperative complications and long-term prognosis of resectable pancreatic cancer after pancreaticoduodenectomy based on CT imaging, and combined with sarcopenia and related clinicopathological features to predict the long-term prognosis, which is expected to improve the postoperative follow-up strategy and long-term survival rate.

## Materials and methods

### Patient selection

**Figure 1:**
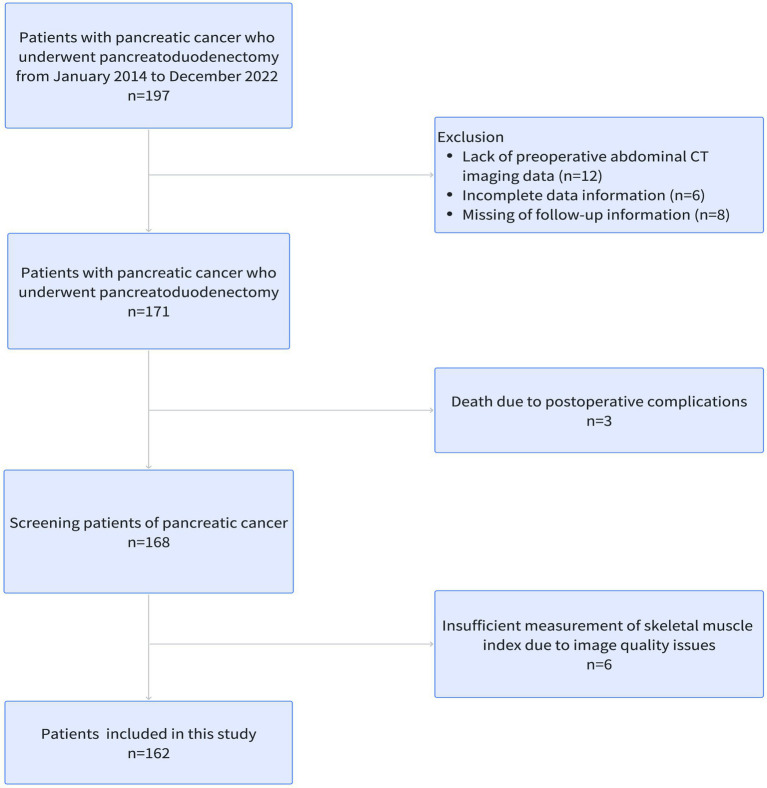
Flowchart of our study.

This retrospective study included patients with pancreatic cancer who underwent pancreaticoduodenectomy in the Department of hepatobiliary pancreatic splenic surgery of Beijing Chao-Yang Hospital from January 2015 to December 2022. The inclusion criteria were as follows: (1) pathological confirmed pancreatic cancer; (2) age ≥ 18 years; (3) absence of preoperative neoadjuvant chemoradiotherapy; and (4) completion of preoperative routine abdominal and pelvic CT. The exclusion criteria were: (1) age < 18 years old; (2) presence of concurrent malignancies; (3) preoperative abdominal CT images were incomplete; and (4) perioperative mortality and follow-up data were incomplete. The flowchart of this study is shown in Figure 1. All the information of patients was confidential, all patients signed informed consent, and this retrospective study was conducted in accordance with the Declaration of Helsinki.

### Data collection and follow-up

Clinical and pathological characteristic variables were obtained from the electronic medical record system, including age, gender, body mass index (BMI), American Society of anesthesiologists (ASA), history of diabetes, smoking, cardiovascular disease, preoperative leukocytes, neutrophils, lymphocytes, monocytes, platelets, hemoglobin, neutrophil to lymphocyte ratio (NLR), prothrombin time (PT), CA199, albumin, creatinine, total bilirubin, direct bilirubin, whether to accept preoperative biliary drainage (PBD), operation time, intraoperative blood loss, postoperative tumor size, differentiation, TNM stage, lymph node invasion, vascular invasion and whether to receive postoperative adjuvant chemotherapy. Short term outcomes included postoperative complications, pancreatic fistula, biliary fistula, bleeding, abdominal infection, and delayed gastric emptying.

We followed up the discharged patients by telephone or outpatient service, and the follow-up date was up to January 2024. The patients were followed up every 3 months and underwent abdominal CT or serum biochemical examination to determine whether the tumor recurred. The primary endpoint of this study was overall survival (OS), which was defined as the period from pancreaticoduodenectomy to death or termination of follow-up. The secondary end point was recurrence-free survival (RFS), which was from postoperative to tumor recurrence, distant metastasis or termination of follow-up.

### Image analysis

**Figure 2:**
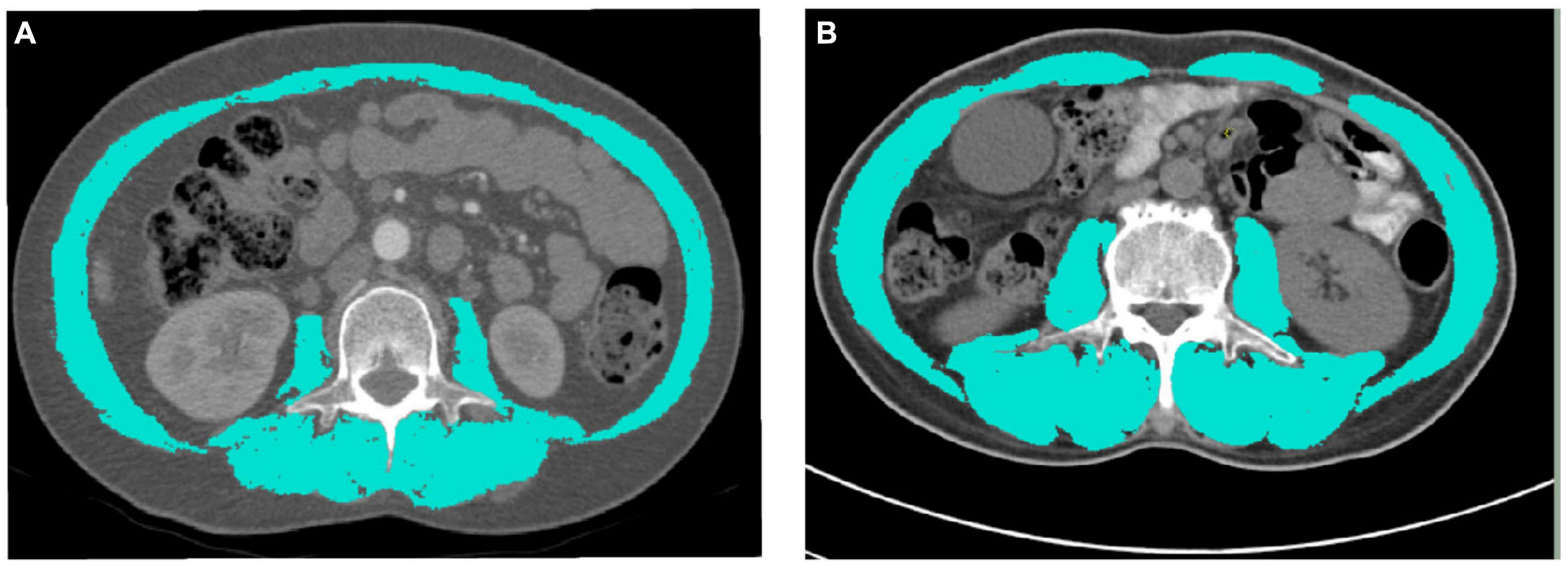
The skeletal muscle index (SMI) of the third lumbar vertebra on CT images. **(A)** The SMI was 34.73cm^2^/m^2^ in a female patient. **(B)** A male patient with a SMI of 48.20cm^2^/m^2^.

Because conventional CT distinguishes muscle tissue and adipose tissue by attenuation of X-ray beam through different tissues and has high resolution, it is increasingly used to evaluate the quantity and quality of muscle in sarcopenia (17). The application of CT in the diagnosis of sarcopenia usually selects a certain part of the cross-section. According to the domestic and foreign literature, the parts measured by CT mainly include the thigh, abdomen and upper arm. The cross-section area (CSA) of skeletal muscle at the level of the third lumbar vertebra (L3) has been proved to be the most relevant part of the whole body measurement, and it is the preferred part for muscle evaluation using CT scan (18). Slice-O-matic V5.0 (Tomovision, Montreal, QC, Canada) software is used to measure the skeletal muscle area (SMA) of the third lumbar vertebra on preoperative non contrast enhanced CT cross-sectional images, including psoas major, quadratus psoas, erector spinae, multifidus, rectus abdominis, transversus abdominis, internal oblique abdominis and external oblique abdominis. According to the Hounsfield scale, tissues ranging from −29 to +150 HU were classified as muscle tissues (Figure 2) (19). Then divide the L3 skeletal muscle area (cm^2^) by the square of height to calculate the skeletal muscle index (SMI) (cm^2^/m^2^). According to the guidelines of the Japanese Society of Hepatology, the standard of sarcopenia (low SMI) is 42 cm^2^/m^2^ for men and 38 cm^2^/m^2^ for women (20). In addition, the sarcopenia criteria proposed by Fujiwara (21) (male 36.2 cm^2^/m^2^, female 29.6 cm^2^/m^2^) and sarcopenia of pancreatic cancer in Asian population (male 42.2 cm^2^/m^2^, female 33.9 cm^2^/m^2^) were also analyzed (22).

### Statistical analysis

Continuous variables are expressed as mean ± standard deviation (normal distribution) with two samples *T*-test or median (interquartile distance) (non-normal distribution) with Mann–Whitney U test. The categorical variables were analyzed as percentages and compared using Pearson’s chi-square test or Fisher’s exact test. Kaplan-Meier curves with the log-rank test were used to survival analysis. Logistic regression analyses were performed to analyze the association between sarcopenia and complications after PD. Variables with statistical significance (*p* < 0.05) in univariate logistic regression were included in multivariate regression analysis. Cox proportional hazards regression analysis with hazard ratios (HRs) and corresponding 95% confidence intervals (CIs) were performed to evaluate the correlation between long-term prognosis (overall survival and recurrence-free survival) and variables (clinicopathological characteristics and sarcopenia) in patients with PC. The variables with *p* < 0.05 in univariate Cox regression were included in multivariate regression analysis. Receiver operating characteristic (ROC) curves with the area under the curve (AUC) were performed to assess the predictive performance of the clinicopathological characteristics alone and with sarcopenia models. All analyses were conducted using R (version 4.1.2), and *p*-value <0.05 is considered statistically significant.

## Results

### Patient characteristics

Our study retrospectively analyzed 162 patients with PC who underwent PD (mean age, 63.78 ± 10.27 years, 92 males and 70 females). Of these patients, 83 (51%) had preoperative sarcopenia in Japanese Society of Hepatology criteria. In the Asian pancreatic cancer population criteria and Fujiwara criteria, sarcopenia and non-sarcopenia were 67 (41%), 95 (59%) and 19 (12%), 143 (88%), respectively. The median SMI in non-sarcopenia group and sarcopenia group were 42.8 cm^2^/m^2^ (interquartile 39.6–45.4) and 37.6 cm^2^/m^2^ (interquartile 35.2–40.2), respectively. The mean CA199 of the study cohort was 143.7 U/mL, so we divided it into two groups according to the value. There was no discrepancy between sarcopenia group and non-sarcopenia group in age, gender, BMI, ASA, history of diabetes, smoking, cardiovascular disease, leukocytes, neutrophils, lymphocytes, platelets, hemoglobin, NLR, CA199, PBD, albumin, creatinine, total bilirubin, direct bilirubin, operation time, tumor size, surgery method (158 open pancreaticoduodenectomy, OPD and 4 laparoscopic pancreaticoduodenectomy, LPD), TNM stage, lymph node metastasis, vascular invasion and postoperative chemotherapy (*p* > 0.05). In the total cohort, R0 resection and R1 resection were 10 (6%) and 152 (94%), respectively. R0 resection and R1 resection were 77 (93%), 75 (95) and 6 (7%), 4 (5%) in sarcopenia group and non-sarcopenia group, respectively (*p* = 0.7). Notably, compared with the non-sarcopenia group, the sarcopenia group had higher monocytes (0.45 ± 0.17 versus 0.39 ± 0.14), more surgery bleeding (median 600.00 versus 500) and poor differentiation proportion (36% versus 22%) (Table 1).

**Table 1:** Correlation of sarcopenia and clinicopathological characteristics in patients with pancreatic cancer undergone pancreaticoduodenectomy.

Variables	Overall (*N* = 162)	Non-Sarcopenia (*N* = 79)	Sarcopenia (*N* = 83)	*p* value
Age (Years)	63.78 (10.27)	62.94 (10.31)	64.59 (10.24)	0.2
Gender				0.3
Male	92 (57%)	48 (61%)	44 (53%)	
Female	70 (43%)	31 (39%)	39 (47%)	
BMI (Kg/m^2^)	22.93 (2.75)	23.21 (2.97)	22.66 (2.53)	0.4
ASA				0.3
I–II	86 (53%)	45 (57%)	41 (49%)	
III–IV	76 (47%)	34 (43%)	42 (51%)	
Diabetes				0.7
None	115 (71%)	55 (70%)	60 (72%)	
Yes	47 (29%)	24 (30%)	23 (28%)	
Smoking				0.8
None	107 (66%)	53 (67%)	54 (65%)	
Yes	55 (34%)	26 (33%)	29 (35%)	
Cardiovascular disease				0.14
None	93 (57%)	50 (63%)	43 (52%)	
Yes	69 (43%)	29 (37%)	40 (48%)	
Leukocyte (×109/L)	6.28 (2.21)	5.98 (1.94)	6.57 (2.42)	0.14
Neutrophils (×109/L)	4.16 (2.07)	3.93 (1.81)	4.38 (2.27)	0.2
Lymphocyte (×109/L)	1.49 (0.55)	1.45 (0.46)	1.53 (0.63)	0.7
Monocyte (×109/L)	0.42 (0.16)	0.39 (0.14)	0.45 (0.17)	0.034
Platelet (×109/L)	224.04 (81.44)	216.00 (67.74)	231.69 (92.39)	0.5
Hemoglobin (×109/L)	120.25 (18.23)	120.99 (16.54)	119.54 (19.78)	0.6
NLR	2.68 (1.91, 3.73)	2.52 (1.92, 3.45)	2.83 (1.93, 4.06)	0.4
PT (S)	12.14 (1.33)	12.09 (1.19)	12.18 (1.46)	0.8
CA199 (U/ml)	143.70 (33.80, 556.13)	152.70 (45.30, 550.10)	133.40 (32.95, 557.00)	0.5
PBD				0.1
None	111 (69%)	59 (75%)	52 (63%)	
Yes	51 (31%)	20 (25%)	31 (37%)	
Albumin (g/L)	37.91 (4.67)	38.22 (4.67)	37.61 (4.67)	0.3
Creatinine (μmol/L)	61.23 (18.99)	61.67 (14.99)	60.81 (22.22)	0.14
TB (μmol/L)	58.95 (12.63, 143.53)	47.70 (13.25, 145.40)	61.10 (12.50, 137.25)	0.7
DB (μmol/L)	45.00 (4.83, 115.65)	37.00 (5.40, 116.20)	51.40 (4.45, 115.60)	0.8
Surgery method				0.9
OPD	158 (98%)	77 (97%)	81 (98%)	
LPD	4 (2%)	2 (3%)	2 (2%)	
Surgery time (h)	11.29 (3.39)	11.16 (3.68)	11.42 (3.10)	0.5
Blood (ml)	500.00 (400.00, 800.00)	500.00 (400.00, 800.00)	600.00 (450.00, 1,000.00)	0.039
Tumor size (cm)				0.4
≤3	77 (48%)	40 (51%)	37 (45%)	
>3	85 (52%)	39 (49%)	46 (55%)	
Differentiation				0.04
Poor	47 (29%)	17 (22%)	30 (36%)	
Well/Moderate	115 (71%)	62 (78%)	53 (64%)	
TNM				0.5
I	53 (33%)	28 (35%)	25 (30%)	
II	79 (49%)	39 (49%)	40 (48%)	
III	30 (19%)	12 (15%)	18 (22%)	
Resection margin				0.7
R1	10 (6%)	4 (5%)	6 (7%)	
R0	152 (94%)	75 (95%)	77 (93%)	
Lymph metastasis				0.2
None	60 (37%)	33 (42%)	27 (33%)	
Yes	102 (63%)	46 (58%)	56 (67%)	
Vascular invasion				0.4
None	73 (45%)	38 (48%)	35 (42%)	
Yes	89 (55%)	41 (52%)	48 (58%)	
Chemotherapy				0.8
None	73 (45%)	35 (44%)	38 (46%)	
Yes	89 (55%)	44 (56%)	45 (54%)	

### Discrepancy of tumor recurrence between sarcopenia group and non-sarcopenia group

We analyzed the postoperative tumor recurrence of sarcopenia group and non-sarcopenia group, and found that the tumor recurrence rate of sarcopenia group was higher than that of non-sarcopenia group (75% versus 59%, *p* = 0.039) (Table 2). The common site of recurrence after PD in PC patients was liver, which was 28 cases and 29 cases in sarcopenia group and non-sarcopenia group, followed by abdomina, omentum, lymph node, stomach, and colon, which were 20 and 13 cases, respectively, and then lung and bone, which were 5 and 3, 3, and 0 cases, respectively. There were 9 cases and 3 cases of recurrence at other sites in the two groups (Table 2). After that, we divided the study cohort into recurrence group (*n* = 109) and non-recurrence group (*n* = 53), and compared the differences in clinicopathological characteristics between the two groups. Compared with the no- recurrence group, the average age of the recurrence group was slightly lower (62.55 ± 10.55 versus 66.32 ± 9.26, *p* = 0.033), while the mean albumin was slightly higher (38.41 ± 4.78 versus 36.89 ± 4.30, *p* = 0.045) (Table 3). Notably, the proportion of tumor size >3 (59% versus 40%, *p* = 0.022), TNM stage II–III (83% versus 36%, *p* < 0.001), and postoperative pathological lymph node metastasis (80% versus 28%, *p* < 0.001) in the recurrence group were higher than those in the non-recurrence group (Table 3). There was no significant difference in other clinicopathological variables between the two groups.

**Table 2:** Effect of sarcopenia on the recurrence of patients in pancreatic cancer after pancreaticoduodenectomy.

	All cases (*N* = 162)	Non-Sarcopenia (*N* = 79)	Sarcopenia (*N* = 83)	*p* value
Recurrence	109(67%)	47 (59%)	62 (75%)	0.039
Liver	57	29	28	
Abdominal/Omentum/Lymph node/Stomach/Colon	33	13	20	
Lung	8	3	5	
Bone	3	0	3	
Others	12	3	9	

**Table 3:** Clinicopathological characteristics of recurrence and non-recurrence in pancreatic cancer patients after pancreaticoduodenectomy.

Variables	Non-recurrence (*N* = 53)	Recurrence (*N* = 109)	*p* value
Age (Years)	66.32 (9.26)	62.55 (10.55)	0.033
Gender			0.9
Male	30 (57%)	62 (57%)	
Female	23 (43%)	47 (43%)	
BMI (Kg/m^2^)	23.41 (3.26)	22.70 (2.46)	0.3
ASA			0.2
I–II	24 (45%)	62 (57%)	
III–IV	29 (55%)	47 (43%)	
Diabetes			0.3
None	35 (66%)	80 (73%)	
Yes	18 (34%)	29 (27%)	
Smoking			0.5
None	37 (70%)	70 (64%)	
Yes	16 (30%)	39 (36%)	
Cardiovascular disease			0.8
None	31 (58%)	62 (57%)	
Yes	22 (42%)	47 (43%)	
Leukocyte (×109/L)	6.24 (1.56)	6.30 (2.48)	0.5
Neutrophils (×109/L)	4.17 (1.45)	4.15 (2.31)	0.3
Lymphocyte (×109/L)	1.47 (0.54)	1.50 (0.56)	0.9
Monocyte (×109/L)	0.43 (0.18)	0.42 (0.15)	0.7
Platelet (×109/L)	224.75 (65.46)	223.69 (88.45)	0.5
Hemoglobin (×109/L)	117.28 (18.81)	121.69 (17.85)	0.2
NLR	2.90 (1.99, 4.03)	2.66 (1.90, 3.65)	0.4
PT (S)	12.21 (1.16)	12.10 (1.41)	0.3
CA199 (U/ml)	109.70 (30.70, 556.40)	150.20 (35.30, 555.30)	0.8
PBD			0.2
None	33 (62%)	78 (72%)	
Yes	20 (38%)	31 (28%)	
Albumin (g/L)	36.89 (4.30)	38.41 (4.78)	0.045
Creatinine (μmol/L)	57.32 (12.75)	63.13 (21.16)	0.2
TB (μmol/L)	47.70 (12.30, 136.80)	59.90 (13.00, 147.00)	0.6
DB (μmol/L)	30.20 (4.90, 111.50)	46.20 (4.60, 119.10)	0.8
Surgery time (h)	11.03 (3.37)	11.42 (3.40)	0.4
Blood (ml)	600.00 (400.00, 800.00)	500.00 (400.00, 800.00)	0.8
Tumor size (cm)			0.022
≤3	32 (60%)	45 (41%)	
>3	21 (40%)	64 (59%)	
Differentiation			0.11
Poor	11 (21%)	36 (33%)	
Well/Moderate	42 (79%)	73 (67%)	
TNM			<0.001
I	34 (64%)	19 (17%)	
II	16 (30%)	63 (58%)	
III	3 (6%)	27 (25%)	
Lymph metastasis			<0.001
None	38 (72%)	22 (20%)	
Yes	15 (28%)	87 (80%)	
Vascular invasion			0.5
None	26 (49%)	47 (43%)	
Yes	27 (51%)	62 (57%)	
Chemotherapy			0.9
None	24 (45%)	49 (45%)	
Yes	29 (55%)	60 (55%)	

### Correlation between sarcopenia and short-term postoperative outcomes in Japanese Society of Hepatology sarcopenia criteria

We analyzed the postoperative complications after PD in sarcopenia group and non-sarcopenia group. At present, Clavein-Dindo classification system has become a perfect system to define the postoperative complications and severity (23). There was no significant difference in the incidence of Clavein-Dindo I–II (28% versus 23%, *p* = 0.5), Clavein-Dindo III (13% versus 8%, *p* = 0.2) and Clavein-Dindo IV (2% versus 1%, *p* = 0.9) complications between sarcopenia group and non-sarcopenia group (Table 4). According to the International Study Group of Pancreatic Surgery (ISGPS) (24), the types of postoperative complications of pancreatic cancer were analyzed, including pancreatic fistula, biliary fistula, abdominal bleeding, abdominal infection and delayed gastric emptying. The incidence of pancreatic fistula was 20% (17/83) in the sarcopenia group and 10% (8/79) in the non-sarcopenia group (*p* = 0.068). The incidence of biliary fistula was 7% (6/83) in the sarcopenia group and 4% (3/79) in the non-sarcopenia group (*p* = 0.5). The incidence of intra-abdominal bleeding was 12% (10/83) in the sarcopenia group and 8% (6/79) in the non-sarcopenia group (*p* = 0.3). The overall rate of intra-abdominal infection was 16% (13/83) in the sarcopenia group and 8% (6/79) in the non-sarcopenia group (*p* = 0.073). The overall rate of delayed gastric emptying was 11% (9/83) in the sarcopenia group and 10% (8/79) in the non-sarcopenia group (*p* = 0.9) (Table 4). No significant differences were observed between the two groups. Further, we analyzed the difference of postoperative complications between OPD group and LPD group, and found that there was no significant discrepancy in the incidence of Clavein-Dindo I–II, Clavein-Dindo III, Clavein-Dindo IV, pancreatic fistula, biliary fistula, abdominal bleeding, infection and delayed gastric emptying between the two groups (Supplementary Table S1) (all *p* > 0.05).

**Table 4:** Postoperative complications and outcomes in pancreatic cancer patients with sarcopenia compared to non-sarcopenia.

Variables	Non-Sarcopenia (*N* = 79)	Sarcopenia (*N* = 83)	*p* value
Clavien-Dindo I–II			0.5
None	61 (77%)	60 (72%)	
Yes	18 (23%)	23 (28%)	
Clavien-Dindo III			0.2
None	73 (92%)	72 (87%)	
Yes	6 (8%)	11 (13%)	
Clavien-Dindo IV			0.9
None	78 (99%)	81 (98%)	
Yes	1 (1%)	2 (2%)	
Pancreatic fistula			0.068
None	71 (90%)	66 (80%)	
Yes	8 (10%)	17 (20%)	
Biliary fistula			0.5
None	76 (96%)	77 (93%)	
Yes	3 (4%)	6 (7%)	
Intra-abdominal bleeding			0.3
None	73 (92%)	73 (88%)	
Yes	6 (8%)	10 (12%)	
Infection			0.11
None	73 (92%)	70 (84%)	
Yes	6 (8%)	13 (16%)	
Delayed gastric emptying			0.9
None	71 (90%)	74 (89%)	
Yes	8 (10%)	9 (11%)	

In order to eliminate confounding factors and further determine the related risk factors of complications after PD in PC, logistic regression analysis was performed. The results suggested that sarcopenia was not the influencing factor of postoperative complications, while the albumin ≤35 (albumin >35 versus albumin ≤35, OR = 0.43, 95% CI: 0.21–0.86, *p* = 0.02), lymph metastasis (OR = 3.70, 95% CI: 1.76–7.77, *p* < 0.001) and TNM stage (OR = 2.79, 95% CI: 1.32–5.88, *p* = 0.01) was risk factors for postoperative complications in univariate logistic regression analysis (Table 5). Then we included the *p* < 0.05 covariate in univariate logistic regression analysis into multivariate logistic regression analysis to determine the related risk factors of postoperative complications. The results suggested that the albumin ≤35 (albumin >35 versus albumin ≤35, OR = 0.36, 95% CI: 0.17–0.76, *p* = 0.007) and lymph metastasis (OR = 3.63, 95% CI: 1.25–10.57, *p* = 0.02) was independent risk variables for postoperative complications (Table 5).

**Table 5:** Univariate and multivariate analysis of variables that predict overall complications in patients of pancreatic cancer undergone pancreaticoduodenectomy.

Variables	Univariate regression	*p* value	Multivariate regression	*p* value
	OR (95% CI)		OR (95% CI)	
Age (Years)		0.12		
≤65	Reference			
>65	0.60 (0.31–1.15)			
Gender		0.15		
Female	Reference			
Male	1.61 (0.84–3.08)			
BMI (Kg/m^2^)		0.56		
≤24	Reference			
>24	0.82 (0.41–1.61)			
ASA		0.44		
I–II	Reference			
III–IV	1.29(0.68–2.43)			
Diabetes		0.64		
None	Reference			
Yes	1.18 (0.59–2.36)			
Albumin (g/L)		0.02		0.007
≤35	Reference		Reference	
>35	0.43 (0.21–0.86)		0.36 (0.17–0.76)	
CA199 (U/ml)		0.87		
≤143.7	Reference			
>143.7	1.05 (0.56–1.99)			
PBD		0.78		
None	Reference			
Yes	1.1 (0.56–2.18)			
Surgery method				
OPD	Reference	0.6		
LPD	0.54 (0.06–5.35)			
Differentiation		0.64		
Well/Moderate	Reference			
Poor	1.18 (0.59–2.36)			
Tumor size		0.2		
≤3.0	Reference			
>3.0	1.53 (0.80–2.91)			
Lymph metastasis		<0.001		0.02
None	Reference		Reference	
Yes	3.70 (1.76–7.77)		3.63 (1.25–10.57)	
Vascular invasion		0.87		
None	Reference			
Yes	0.95 (0.50–1.79)			
TNM Stage		0.01		
I	Reference		Reference	0.7
II–III	2.79 (1.32–5.88)		1.23 (0.42–3.67)	
Chemotherapy		0.25		
None	Reference			
Yes	0.69 (0.36–1.31)			
Sarcopenia		0.12		
None	Reference			
Yes	2.83 (1.46–5.47)			
NLR	1.65 (0.87–3.15)	0.29		

### Correlation between sarcopenia and postoperative outcomes in Asian pancreatic cancer population and Fujiwara sarcopenia criteria

According to pancreatic cancer in Asian population criteria (sarcopenia = 67, non-sarcopenia = 95) and Fujiwara criteria (sarcopenia = 19, non-sarcopenia = 143), we performed univariate and multivariate analysis on the risk factors affecting the complications after PD. In the Asian pancreatic cancer population criteria, univariate logistic analysis revealed that factors such as albumin ≤35 (albumin >35 versus albumin ≤35, OR = 0.43, 95% CI: 0.21–0.86, *p* = 0.02), lymph metastasis (OR = 3.70, 95% CI: 1.76–7.77, *p* < 0.001), advanced TNM stage (OR = 2.79, 95% CI: 1.32–5.88, *p* = 0.01) and sarcopenia (OR = 2.08, 95% CI: 1.09–3.98, *p* = 0.03) were associated with an increased risk of postoperative complications in PC patients. Multivariate logistic further confirmed that aluminum ≤35 (albumin >35 versus albumin ≤35, OR = 0.38, 95% CI: 0.18–0.80, *p* = 0.01) and lymph metastasis (OR = 3.53, 95% CI: 1.17–10.64, *p* = 0.02) remained significant risk factors for postoperative complications (Supplementary Table S2).

In Fujiwara criteria, univariate logistic analysis showed that albumin ≤35 (albumin >35 versus albumin ≤35, OR = 0.43, 95% CI: 0.21–0.86, *p* = 0.002), tumor size >3.0 cm (OR = 2.79, 95% CI: 1.32–5.88, *p* = 0.01), lymph metastasis (OR = 3.70, 95% CI: 1.76–7.77, *p* < 0.001) and advanced TNM stage (OR = 2.79, 95% CI: 1.32–5.88, *p* = 0.01) were related risk factors of postoperative complications in PC patients. Further, multivariate analysis showed that the independent risk factors for postoperative complications in PC were albumin ≤35 (albumin >35 versus albumin ≤35, OR = 0.36, 95% CI: 0.17–0.76, *p* = 0.007) and lymph metastasis (OR = 3.63, 95% CI: 1.25–10.57, *p* = 0.02) (Supplementary Table S3). There was no correlation between sarcopenia, surgical methods (OPD or LPD) and complications after PD in the above three criteria.

### Kaplan-Meier curves in different subgroups

**Figure 3:**
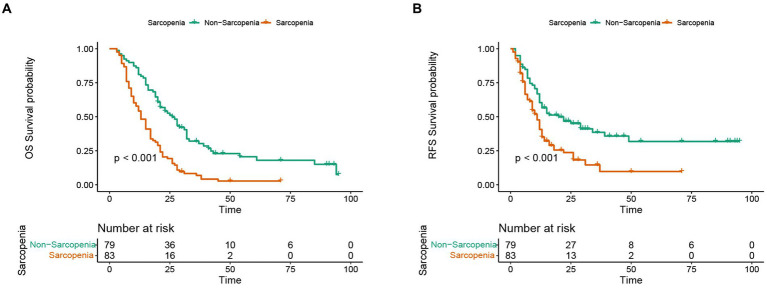
Discrepancy in Kaplan-Meier curve for long-term prognosis between sarcopenia group and non-sarcopenia group. **(A)** Kaplan-Meier curve in overall survival between sarcopenia group and non-sarcopenia group. **(B)** Kaplan-Meier curve in recurrence-free survival between sarcopenia group and non-sarcopenia group.

**Figure 4:**
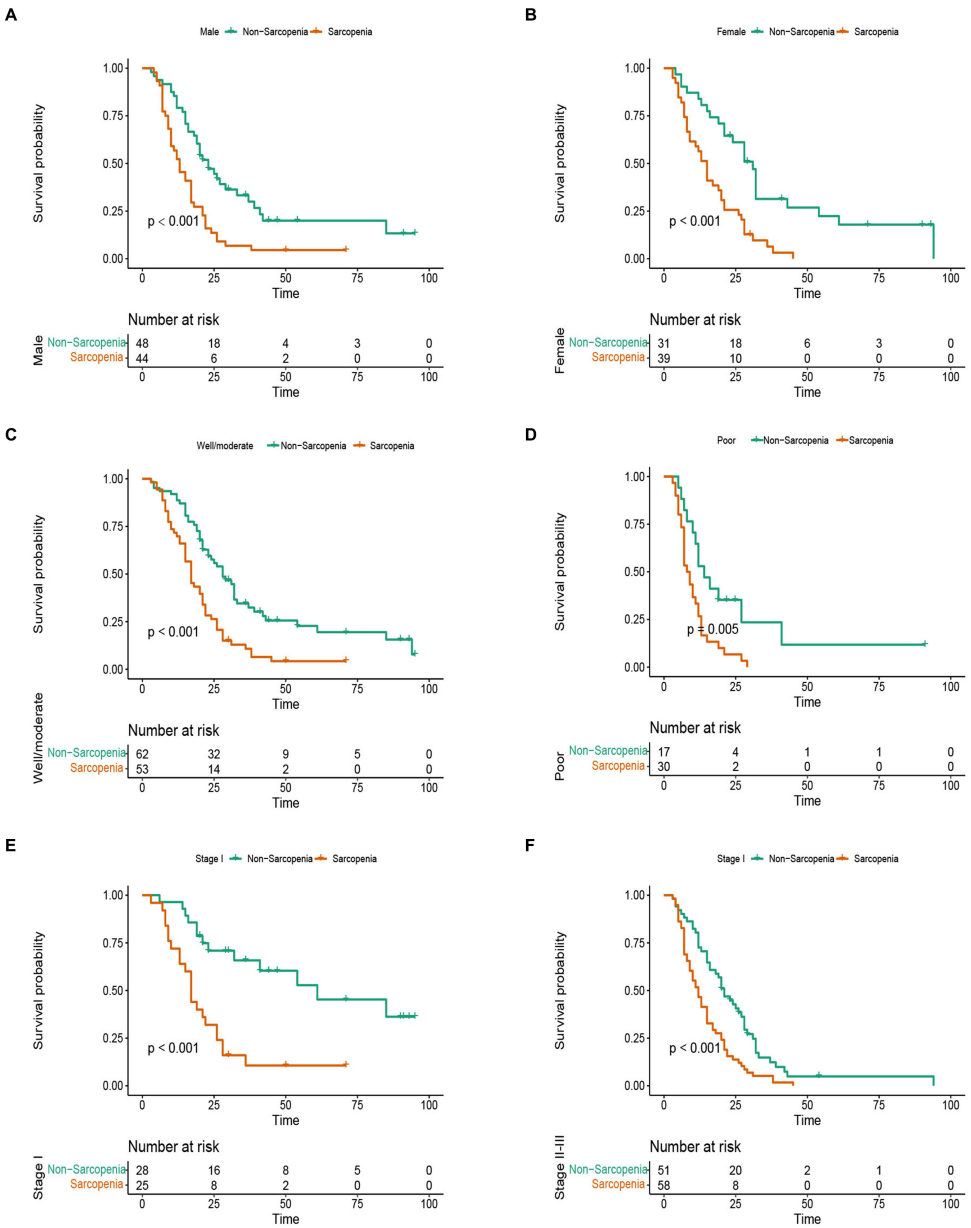
Stratified analysis of overall survival in sarcopenia group and non-sarcopenia group. **(A)** Kaplan-Meier curve of male patients with sarcopenia and non-sarcopenia. **(B)** Kaplan-Meier curve of female patients with sarcopenia and non-sarcopenia. **(C)** Kaplan-Meier curve of well/moderate differentiation with sarcopenia and non-sarcopenia. **(D)** Kaplan-Meier curve of poor differentiation with sarcopenia and non-sarcopenia. **(E)** Kaplan-Meier curve of stage I with sarcopenia and non-sarcopenia. **(F)** Kaplan-Meier curve of stage II–III with sarcopenia and non-sarcopenia.

**Figure 5:**
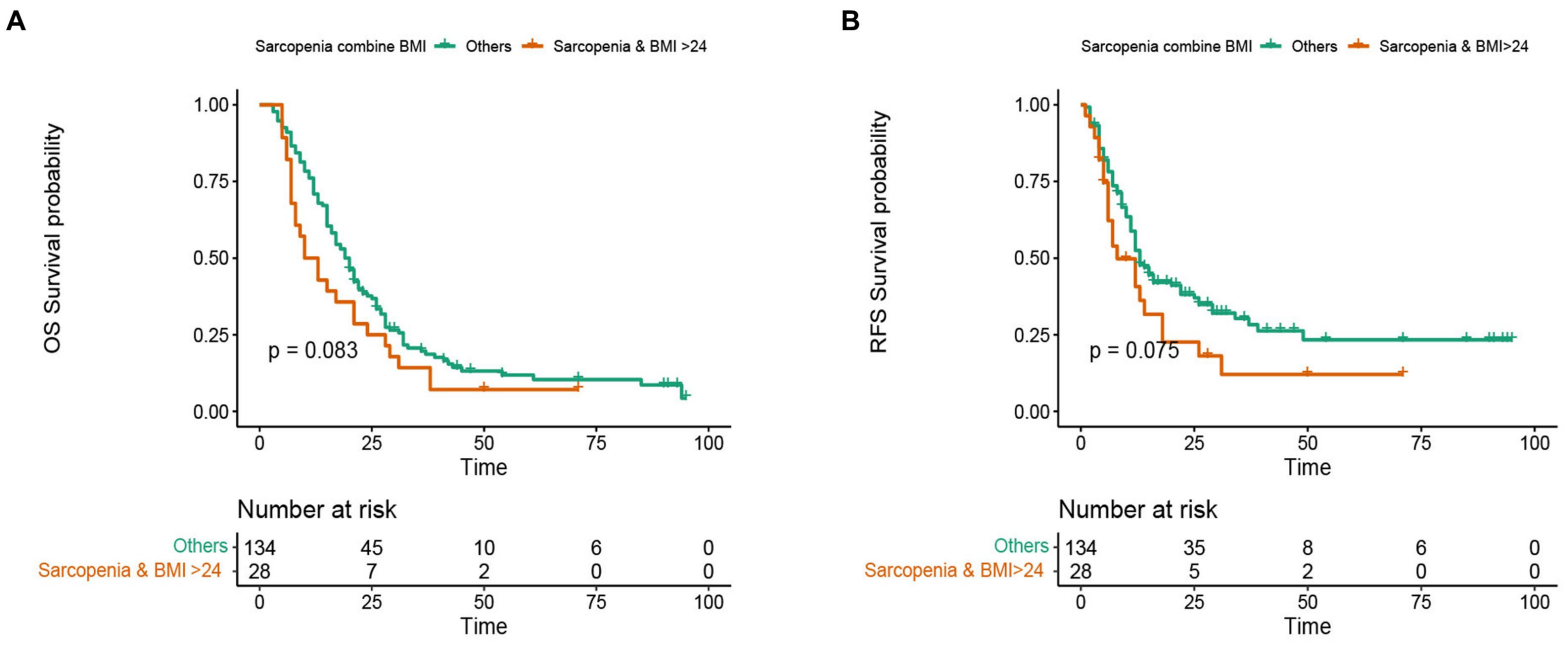
Kaplan-Meier curve of subgroup analysis consisting of sarcopenia and higher BMI. **(A)** Kaplan-Meier curve for overall survival of sarcopenia group combined with high BMI group and other groups. **(B)** Kaplan-Meier curve for recurrence free survival of sarcopenia group combined with high BMI group and other groups.

In this study, the median OS of the whole cohort was 18.5 months, and the median RFS was 13 months. The survival discrepancy of OS and RFS between sarcopenia group and non-sarcopenia group were analyzed. The results indicated that the sarcopenia group had a significantly shorter OS compared to the non-sarcopenia group (median 13 months versus 26 months, *p* < 0.001) (Figure 3A). Similarly, the RFS in the sarcopenia group was lower than that in the non-sarcopenia group (median 11 months versus 20 months, *p* < 0.001) (Figure 3B). Additionally, differences in long-term survival between the sarcopenia and non-sarcopenia groups were further analyzed within various subgroups. In the male population, there were 44 sarcopenia and 48 non-sarcopenia, respectively. Kaplan-Meier curve with log-rank test showed that the OS of non-sarcopenia group was significantly longer than that of sarcopenia group (median 31 months versus 15 months, *p* < 0.001) (Figure 4A). Similarly, in the female population, the OS of the non-sarcopenia group was also significantly longer than that of the sarcopenia group (median 23 months versus 13 months, *p* < 0.001) (Figure 4B). A stratified analysis was conducted based on tumor differentiation and TNM stage. For well/moderate differentiation, OS in the sarcopenia group (median 17 months versus 28 months, *p <* 0.001) was significantly lower than in the non-sarcopenia group. For poor differentiation, OS of sarcopenia group was also lower than that of non-sarcopenia group (median 8.5 months versus 14 months, *p =* 0.005) (Figures 4C,D). For stage I, OS in sarcopenia patients was remarkable lower than in non-sarcopenia (median 17 months versus 61 months, *p* < 0.001). Similarly, there was a notable difference in the survival rate of patients with stage II–III in sarcopenia group and non-sarcopenia group (median 12 months versus 21 months, *p* < 0.001) (Figures 4E,F). Then we combined BMI and sarcopenia, divided the patients into Sarcopenia & BMI > 24 group and other group, and compared the overall survival and recurrence free survival of the two groups. Kaplan-Meier curve with log rank test showed that there was no significant difference in overall survival (*p* = 0.083) and recurrence free survival (*p* = 0.075) between Sarcopenia & BMI > 24 group and other group (Figures 5A,B).

### Variables correlated with OS and RFS in Japanese Society of Hepatology sarcopenia criteria

We performed univariate and multivariate Cox regression analysis to identify the variables affecting the long-term prognosis of PC after PD. Univariate Cox regression analysis showed that CA199 > 143.7 U/mL (HR = 1.78, 95% CI: 1.27–2.50, *p* < 0.001), poor differentiation (HR = 2.53, 95 CI%:1.74–3.66, *p* < 0.001), tumor diameter > 3 cm (HR = 1.77, 95% CI: 1.26–2.48, *p* = 0.001), lymph node metastasis (HR = 1.60, 95% CI: 1.12–2.28, *p* = 0.01), vascular invasion (HR = 1.46, 95% CI: 1.04–2.05, *p* = 0.028), advanced TNM stage (HR = 2.51, 95% CI: 1.69–3.72, *p* < 0.001) and sarcopenia (HR = 2.53, 95% CI: 1.78–3.58, *p* < 0.001) were independent risk factors for long-term overall survival in patients with PC. Multivariate Cox analysis showed that sarcopenia was an independent risk factor for OS (HR = 2.49, 95% CI: 1.73–3.60, *p* < 0.001) in patients with PC. In addition, other independent risk clinicopathological variables affecting OS included CA199 > 143.7 U/mL (HR = 1.51, 95% CI: 1.07–2.10, *p* = 0.02), poor differentiation (HR = 2.19, 95% CI: 1.49–3.20, *p* < 0.001), tumor diameter > 3 cm (HR = 1.51, 95% CI: 1.04–2.20, *p* = 0.031) and advanced TNM stage (HR = 2.19, 95% CI: 1.21–3.90, *p* = 0.01) (Table 6). The univariate Cox regression analysis showed that poor differentiation (HR = 2.74, 95% CI: 1.81–4.13, *p* < 0.001), tumor diameter > 3 cm (HR = 1.82, 95% CI: 1.24–2.68, *p* = 0.002), lymph node metastasis (HR = 3.46, 95% CI: 2.16–5.56, *p* < 0.001), advanced TNM stage (HR = 3.96, 95% CI: 2.37–6.60, *p* < 0.001) and sarcopenia (HR = 1.96, 95% CI: 1.33–2.88, *p* < 0.001) were independent risk factors for recurrence free survival in patients with PC. After multivariate Cox analysis, sarcopenia is also an independent risk factor for RFS (HR = 1.70, 95% CI: 1.12–2.50, *p* = 0.012), other independent risk factors affecting RFS included poor differentiation (HR = 2.40, 95% CI: 1.58–3.70, *p* < 0.001), lymph metastasis (HR = 2.00, 95% CI: 1.08–3.90, *p* = 0.027) and advanced TNM stage (HR = 2.20, 95% CI: 1.08–4.40, *p* = 0.031) (Table 7).

**Table 6:** Univariate and multivariate Cox regression analysis of clinicopathologic variables related to overall survival in patients with pancreatic cancer after pancreaticoduodenectomy.

Variables	Univariate Cox regression	*p* value	Multivariate Cox regression	*p* value
	HR (95% CI)		HR (95% CI)	
Age (years)		0.61		
≤65	Reference			
>65	1.09 (0.78–1.53)			
Gender		0.74		
Female	Reference			
Male	1.06 (0.76–1.48)			
BMI (Kg/m^2^)		0.19		
≤24	Reference			
>24	0.79 (0.55–1.13)			
ASA		0.58		
I–II	Reference			
III–IV	0.91 (0.65–1.27)			
Diabetes		0.28		
None	Reference			
Yes	0.81 (0.56–1.18)			
Albumin		0.76		
≤35	Reference			
>35	1.06 (0.73–1.53)			
CA199		<0.001		0.02
≤143.7	Reference		Reference	
>143.7	1.78 (1.27–2.50)		1.51 (1.07–2.10)	
PBD		0.37		
None	Reference			
Yes	0.85 (0.59–1.22)			
Differentiation		<0.001		<0.001
Well/Moderate	Reference		Reference	
Poor	2.53 (1.74–3.66)		2.19 (1.49–3.20)	
Tumor size		0.001		0.031
≤3.0	Reference		Reference	
>3.0	1.77 (1.26–2.48)		1.51 (1.04–2.20)	
Lymph metastasis		0.01		0.476
None	Reference		Reference	
Yes	1.60 (1.12–2.28)		0.83 (0.50–1.40)	
Vascular invasion		0.028		0.564
None	Reference		Reference	
Yes	1.46 (1.04–2.05)		1.11 (0.78–1.60)	
TNM Stage		<0.001		0.009
I	Reference		Reference	
II–III	2.51 (1.69–3.72)		2.19 (1.21–3.90)	
Chemotherapy				
Gemcitabine	Reference			
FOLFIRINOX + unknown	1.23 (0.75–2.00)	0.41		
None	1.41 (0.97–2.04)	0.07		
Sarcopenia		<0.001		<0.001
None	Reference		Reference	
Yes	2.52 (1.78–3.55)		2.49 (1.73–3.60)	
NLR	0.97 (0.91–1.02)	0.25		

**Table 7:** Univariate and multivariate Cox regression analysis of clinicopathologic variables related to recurrence free survival in patients with pancreatic cancer after pancreaticoduodenectomy.

Variables	Univariate Cox regression	*p* value	Multivariate Cox regression	*p* value
	HR (95% CI)		HR (95% CI)	
Age (Years)		0.42		
≤65	Reference			
>65	0.85 (0.58–1.25)			
Gender		0.68		
Female	Reference			
Male	1.08 (0.74–1.58)			
BMI (Kg/m^2^)		0.16		
≤24	Reference			
>24	0.75 (0.50–1.12)			
ASA		0.24		
I–II	Reference			
III–IV	0.80 (0.54–1.16)			
Diabetes		0.22		
None	Reference			
Yes	0.77 (0.50–1.18)			
Albumin (g/L)		0.16		
≤35	Reference			
>35	1.37 (0.88–2.12)			
CA199 (U/ml)		0.07		
≤143.7	Reference			
>143.7	1.43 (0.98–2.08)			
PBD		0.32		
None	Reference			
Yes	0.81 (0.53–1.23)			
Differentiation		<0.001		<0.001
Well/Moderate	Reference		Reference	
Poor	2.74 (1.81–4.13)		2.40 (1.58–3.70)	
Tumor size (cm)		0.002		0.1
≤3.0	Reference		Reference	
>3.0	1.82 (1.24–2.68)		1.40 (0.94–2.10)	
Lymph metastasis		<0.001		0.027
None	Reference		Reference	
Yes	3.46 (2.16–5.56)		2.0 (1.08–3.90)	
Vascular invasion		0.13		
None	Reference			
Yes	1.35 (0.92–1.97)			
TNM Stage		<0.001		0.031
I	Reference		Reference	
II–III	3.96 (2.37–6.60)		2.20 (1.08–4.40)	
Chemotherapy				
Gemcitabine	Reference			
FOLFIRINOX + Unknown	1.10 (0.63–1.91)	0.74		
None	1.18 (0.78–1.78)	0.44		
Sarcopenia		<0.001		0.012
None	Reference		Reference	
Yes	1.96 (1.33–2.88)		1.70 (1.12–2.50)	
NLR	0.97 (0.91–1.04)	0.34		

### Variables correlated with OS and RFS in Asian pancreatic cancer population and Fujiwara sarcopenia criteria

Similarly, we analyzed the independent factors affecting the long-term OS of PC patients after PD according to the Asian pancreatic cancer population criteria and Fujiwara criteria. Multivariate Cox regression analysis showed that CA199 > 143.7 U/mL (HR = 1.46, 95% CI:1.03–2.10 *p* = 0.035), poor differentiation (HR = 2.21, 95% CI: 1.49–3.30, *p* < 0.001), tumor diameter > 3 cm (HR = 1.61, 95% CI: 1.10–2.40, *p* = 0.013), advanced TNM stage (HR = 2.12, 95% CI: 1.22–4.00, *p* = 0.009) and sarcopenia (HR = 2.59, 95% CI: 1.80–3.70, *p* < 0.001) were independent risk factors for OS after PD in Asian pancreatic cancer population criteria (Supplementary Table S4). Multivariate Cox regression analysis showed that poor differentiation (HR = 2.40, 95% CI:1.59–3.80, *p* < 0.001), lymph metastasis (HR = 2.0, 95% CI: 1.05–3.90, *p* = 0.034), advanced TNM stage (HR = 2.20, 95% CI: 1.07–4.50, *p* = 0.033) and sarcopenia (HR = 2.00, 95% CI: 1.36–3.00, *p* < 0.001) were independent risk factors for RFS after PD (Supplementary Table S5).

According to Fujiwara criteria, the independent risk factors affecting the long-term OS of PC patients after PD were CA199 > 143.7 U/mL (HR = 1.49, 95% CI: 1.05–2.10, *p* = 0.026), poor differentiation (HR = 2.32, 95% CI: 1.57–3.40, *p* < 0.001), tumor diameter > 3 cm (HR = 1.47, 95% CI: 1.01–2.10, *p* = 0.042), advanced TNM stage (HR = 2.17, 95% CI: 1.24–3.80, *p* = 0.007) and sarcopenia (HR = 1.81, 95% CI: 1.08–3.10, *p* = 0.025) (Supplementary Table S6). In terms of RFS, multivariate analysis showed that poor differentiation (HR = 2.50, 95% CI: 1.65–3.90, *p* < 0.001), lymph metastasis (HR = 2.20, 95% CI: 1.17–4.00, *p* = 0.014), and advanced TNM stage (HR = 2.20, 95% CI: 1.12–4.40, *p* = 0.022) were independent risk factors for long-term recurrence free survival in PC (Supplementary Table S7). However, sarcopenia is not associated with long-term RFS of PC after PD.

**Figure 6:**
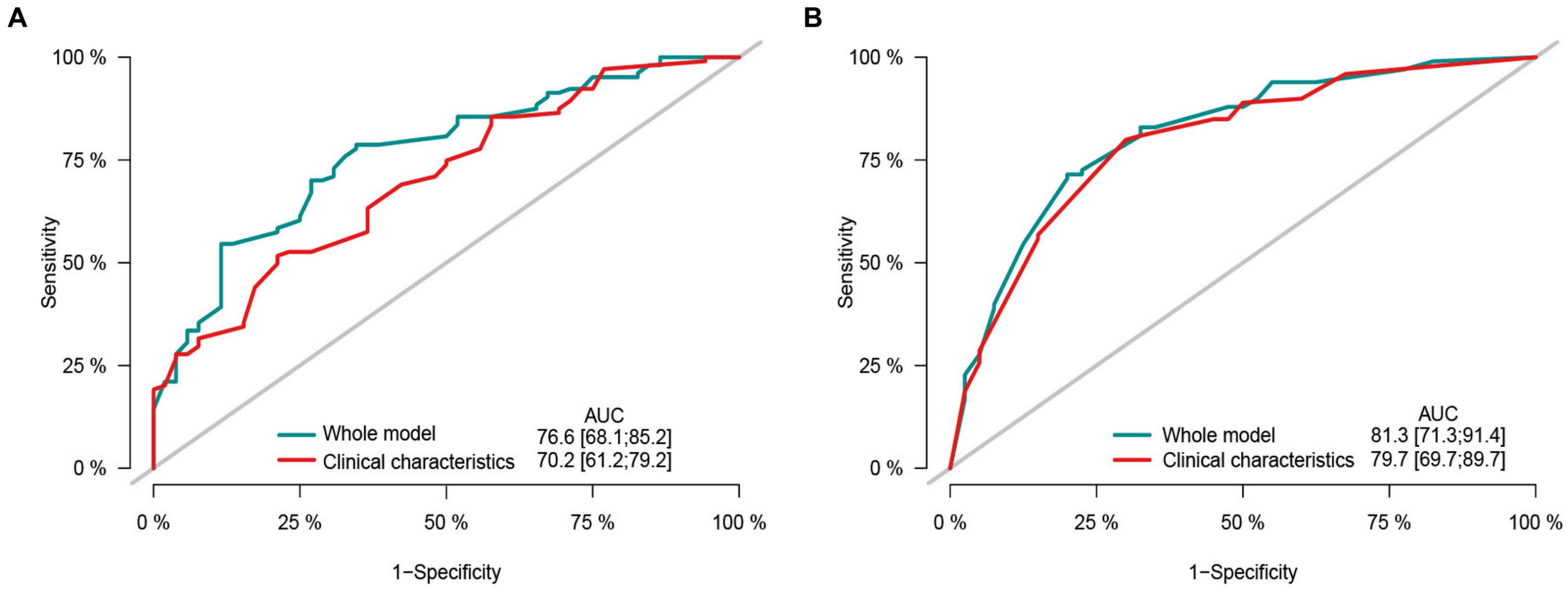
The area under the receiver operating characteristic curve composed by different clinicopathological features and sarcopenia. **(A)** Receiver operating characteristic (ROC) curve by different clinical characteristic in predicting the 2-years overall survival. **(B)** Receiver operating characteristic (ROC) curve by different clinical characteristic in predicting the 2-years recurrence free survival.

The ROC curve analysis demonstrated the predictive performance of the multivariate models for overall survival and recurrence-free survival. The model combined sarcopenia and clinical pathological variables had AUC of 0.766 (95% CI: 0.681–0.852) and 0.813 (95% CI: 0.713–0.914) for both OS and RFS. The AUC of clinicopathological factors model excluding sarcopenia was 0.702 (95% CI: 0.612–0.792) and 0.797 (95% CI, 0.697–0.897) for both OS and RFS (Figures 6A,B). The multivariate models incorporating sarcopenia and clinical variables outperformed the clinical characteristics model.

## Discussion

Our study demonstrates that preoperative sarcopenia is not correlated with the complications of pancreatic cancer after pancreaticoduodenectomy in different Asian sarcopenia criteria, but it is an independent risk factor affecting long-term survival. The predictive performance of sarcopenia combined with clinicopathological characteristics model for long-term survival of PC is superior to clinicopathological factors alone.

Cancer patients are commonly subjected to a variety of tumor specific and non-tumor specific factors, leading to the loss of muscle mass and muscle dysfunction, including age, malnutrition, cancer derived factors, cancer therapy and supportive treatment (25). Sarcopenia can affect the long-term prognosis of a variety of tumors, including oesophageal cancer (26), head and neck tumors (27), malignant lymphoma (28), gastric cancer (29), liver cancer (30). Meanwhile, studies have reported that sarcopenia is associated with postoperative complications in patients with cholangiocarcinoma after hepatectomy (31), which also increases the postoperative complications in patients with gastric cancer (32). In a meta-analysis, there was no statistical discrepancy in the incidence of clinically relevant postoperative pancreatic fistula (7% versus 7, 95% CI: 0.61–1.71; *p* = 0.933), delayed gastric emptying (19% versus 17, 95% CI: 0.80–1.29; *p* = 0.895), abdominal infection (sarcopenic 17% versus 22, 95% CI: 0.75–1.16; *p* = 0.518), Clavien-Dindo grade 3 or above (30% versus 24, 95% CI: 0.86–1.14; *p* = 0.869) in sarcopenia and non-sarcopenia group (33). Similarly, a meta-analysis by Liu et al. (34) shows that sarcopenia is not related to postoperative complications of pancreatic cancer, and similar results were still obtained after subgroup analysis and publication bias. However, in another study, compared with the non-sarcopenia group, the preoperative sarcopenia group had a significantly higher probability of postoperative complications, including Clavien-Dindo classification (*p* = 0.003), surgical site infection (*p* = 0.022) and biliary fistula (*p* = 0.029) (35). Most of the results of the above studies are based on non-Asian groups. In our study, based on a cohort of Chinese pancreatic cancer patients undergone pancreaticoduodenectomy, the results showed that sarcopenia was not an independent risk factor for postoperative complications, including Clavien-Dindo I–IV, pancreatic fistula, biliary fistula, abdominal bleeding, infection and delayed gastric emptying. In addition, after using the Asian pancreatic cancer population criteria (22) and Fujiwara’s sarcopenia criteria (21) to analyze the related independent risk factors of complications in patients with PC after PD, the results showed that sarcopenia was not correlated with postoperative complications. The occurrence of postoperative complications of pancreatic cancer depends more on the trauma of the complex operation itself. Our study provides a reference for exploring the correlation between sarcopenia and postoperative complications in patients with PC after PD.

In several studies, sarcopenia has a far-reaching impact on the long-term survival of PC patients, which can lead to poor long-term survival, whether in the chemotherapy and palliative treatment of unresected pancreatic cancer or resectable pancreatic cancer (36–38). Peng et al. (39) analyzed the long-term impact of preoperative sarcopenia on Chinese pancreatic cancer patients undergoing pancreaticoduodenectomy. The results showed that sarcopenia was an independent risk factor affecting OS, but it did not affect disease free survival (DFS). The research conducted by the authors establishes a robust basis and significant point of reference for investigating the relationship between sarcopenia and the extended survival of PC patients. We also found some limitations. Initially, the study failed to report the outcomes of multivariate analysis on DFS in PC patients. Subsequently, the multivariate analysis revealed that sarcopenia was the sole independent risk factor for OS in PC patients, with clinicopathological factors such as tumor size, grade, and TNM stage having no impact on OS. Some studies have confirmed that clinicopathological factors such as tumor size, grade and TNM stage have different degrees of influence on the long-term prognosis of PC patients after PD (40, 41). Our study used both the Japanese Society of Hepatology and Asian pancreatic cancer population criteria of sarcopenia. Multivariate Cox regression analysis showed that sarcopenia was an independent risk factor affecting the long-term RFS of PC after PD. Similarly, when applying the Fujiwara criteria, only 19 cases of sarcopenia were identified in the sample of 162, and multivariate Cox regression analysis indicated that sarcopenia was not an independent risk factor influencing recurrence-free survival in pancreatic cancer patients. It is suggested that future studies utilizing the Fujiwara criteria should include a sufficiently large sample size to accurately assess the impact of sarcopenia on recurrence-free survival in pancreatic cancer patients following pancreaticoduodenectomy. Zalite’s study showed that sarcopenia was not an independent factor for long term survival in PC patients (12). The variance in research findings may be attributed to the demographic composition of the patient cohorts, with the previous study focusing on western populations while our investigation centers on the prognostic implications of sarcopenia in Chinese PC patients. Furthermore, the above study utilizes a relatively high cut-off value for sarcopenia, as determined by the SMI (38.9 cm^2^/m^2^ for females and less than 55.4 cm^2^/m^2^ for males) at the third lumbar level. In our study, Japanese Society of Hepatology, Asian pancreatic cancer population and Fujiwara sarcopenia criteria were used to systematically elaborate the impact of sarcopenia on short-term complications, long-term OS and RFS of PC after PD. Aoki et al. (42) posited that sarcopenia may not have a significant impact on postoperative complications, but it was identified as an independent risk factor for long-term OS and RFS. Our findings align with this assertion, as demonstrated by our study involving a cohort of Chinese patients diagnosed with PC. Compared with the non-sarcopenia group, the sarcopenia group had a higher postoperative recurrence rate (75% versus 59%, *p* = 0.039), and its long-term overall survival (HR = 2.49, 95% CI: 1.73–3.60, *p* < 0.001) and recurrence free survival (HR = 1.70, 95% CI: 1.12–2.50, *p* = 0.012) were poor. We established a subgroup analysis for patients with PC and found that sarcopenia was associated with poor survival in both male and female patients. Similar results can also be observed in stage I, stage II–III, well/moderate and poor differentiation PC patients. The underlying mechanism may be that sarcopenia affects body functions and then causes metabolic disorders, including imbalance of energy metabolism, heat regulation, insulin sensitivity and amino acid metabolism disorders (43). In addition, skeletal muscle loss often leads to increased intramuscular fat infiltration, which in turn leads to insulin resistance, muscle strength loss and motor dysfunction, which can lead to systolic dysfunction and metabolic and endocrine abnormalities, and ultimately lead to cancer cachexia (44).

High BMI and obesity are another index affecting the long-term prognosis of cancer patients. Sarcopenic obesity is a clinical disease characterized by coexistence of obesity, excessive fat mass (FM) and decreased skeletal muscle mass and function (45). Sarcopenia obesity is becoming more and more common in tumors and chronic diseases. The incidence of sarcopenic obesity is considered to be a new factor closely related to cancer patients, which may lead to deterioration of functional status and shortening of survival time through the interaction of pathophysiological mechanisms (46). In our study, PC patients were divided into sarcopenia & BMI > 24 kg/m^2^ and other groups. Kaplan-Meier curve showed that compared with other groups, the OS and RFS of sarcopenia & BMI > 24 kg/m^2^ were relatively shorter, but there was no significant difference. This may be due to the fact that there are fewer patients with sarcopenia & BMI > 24 kg/m^2^ or obesity. Therefore, a larger sample size study should analyze the survival difference between sarcopenia obesity and non-sarcopenia obesity.

Overall, our results support the argument that sarcopenia needs more attention in patients with PC undergone PD. Radiological examinations, such as CT, provide a convenient and inexpensive opportunity to quickly and accurately determine preoperative sarcopenia. Appropriate and adequate nutritional intervention, functional exercise, and physical function assessment can fully benefit the group with sarcopenia. For the postoperative management of patients with sarcopenia, developing individualized follow-up and management strategies, and maintaining the appropriate increase or balance of skeletal muscle content will help to improve the expected long-term results. Our study also has limitations. First, as a single center retrospective study, the results should be confirmed by a larger cohort of prospective external studies. Secondly, in addition to the preoperative skeletal muscle index evaluated by CT, the dynamic changes of skeletal muscle mass in patients with PC after PD should be further analyzed.

Our study systematically investigated the correlations between preoperative sarcopenia and postoperative complications in PC patients undergone PD and their impact on long-term survival in different Asian criteria. Preoperative sarcopenia does not affect PC complications after PD. The prognosis model combined with sarcopenia and clinicopathological characteristics will further improve the prediction efficiency of long-term prognosis of PC patients. Identifying patients with sarcopenia based on CT images is helpful for clinicians to formulate specific and individualized treatment and follow-up strategies, so as to improve the long-term postoperative results.

## Conclusion

Sarcopenia is not an independent risk factor for complications after PD in patients with PC. Sarcopenia determined by CT is a meaningful parameter to predict the long-term survival of PC patients. Our study emphasizes that the broader attention to the sarcopenia in patients with pancreatic cancer should be performed, in order to carry out risk stratification, preoperative care and postoperative individualized follow-up strategies.

## Data availability statement

The original contributions presented in the study are included in the article/Supplementary material, further inquiries can be directed to the corresponding authors.

## Ethics statement

The studies involving humans were approved by Ethics Committee of Beijing Chaoyang Hospital (No. 2020-D-302). The studies were conducted in accordance with the local legislation and institutional requirements. The participants provided their written informed consent to participate in this study.

## Author contributions

GQ: Data curation, Writing – original draft. CZ: Data curation, Investigation, Writing – original draft. YZ: Data curation, Investigation, Methodology, Writing – review & editing. S-CL: Conceptualization, Formal analysis, Investigation, Supervision, Writing – review & editing. RL: Conceptualization, Investigation, Methodology, Supervision, Writing – review & editing.
