# Supplementary material for: Influence of sarcopenia on postoperative complications and long-term survival in pancreatic cancer patients undergone pancreaticoduodenectomy
Source: Front Nutr. 2024 Jul 4;11:1434630. doi: 10.3389/fnut.2024.1434630 (PMC11254807; doi:10.3389/fnut.2024.1434630)
Supplement: Supplementary file 1 [file Table_1.docx]

Supplementary Tables

Supplementary Table 1 Analysis of the difference of postoperative complications between OPD and LPD

| Variables | OPD (N=158) | LPD (N=4) | P value |
| --- | --- | --- | --- |
| Clavien-Dindo I-II |  |  | 0.9 |
| None | 118 (75%) | 3 (75%) |  |
| Yes | 40 (25%) | 1 (25%) |  |
| Clavien-Dindo III |  |  | 0.9 |
| None | 141 (89%) | 4 (100%) |  |
| Yes | 17 (11%) | 0 (0%) |  |
| Clavien-Dindo IV |  |  | 0.9 |
| None | 155 (98%) | 4 (100%) |  |
| Yes | 3 (1.9%) | 0 (0%) |  |
| Pancreatic fistula |  |  | 0.9 |
| None | 133 (84%) | 4 (100%) |  |
| Yes | 25 (16%) | 0 (0%) |  |
| Biliary fistula |  |  | 0.9 |
| None | 149 (94%) | 4 (100%) |  |
| Yes | 9 (5.7%) | 0 (0%) |  |
| Intra-abdominal bleeding | |  | 0.9 |
| None | 142 (90%) | 4 (100%) |  |
| Yes | 16 (10%) | 0 (0%) |  |
| Infection |  |  | 0.9 |
| None | 139 (88%) | 4 (100%) |  |
| Yes | 19 (12%) | 0 (0%) |  |
| Delayed gastric emptying | |  | 0.4 |
| None | 142 (90%) | 3 (75%) |  |
| Yes | 16 (10%) | 1 (25%) |  |

Supplementary Table 2 Univariate and multivariate analysis of variables that predict overall complications in PC of Asian pancreatic cancer population sarcopenia criteria

| Variables | | Univariate regression | | P value | |  | | Multivariate regression | | P value | |
| --- | --- | --- | --- | --- | --- | --- | --- | --- | --- | --- | --- |
|  | | OR (95% CI ) | |  | |  | | OR (95% CI ) | |  | |
| Age (Years) | |  | | 0.12 | |  | |  | |  | |
| ≤65 | | Reference | |  | |  | |  | |  | |
| ＞65 | | 0.60 (0.31-1.15) | |  | |  | |  | |  | |
| Gender | |  | | 0.15 | |  | |  | |  | |
| Female | | Reference | |  | |  | |  | |  | |
| Male | | 1.61 (0.84-3.08) | |  | |  | |  | |  | |
| BMI (Kg/m2) | |  | | 0.56 | |  | |  | |  | |
| ≤24 | | Reference | |  | |  | |  | |  | |
| ＞24 | | 0.82 (0.41-1.61) | |  | |  | |  | |  | |
| ASA | |  | | 0.44 | |  | |  | |  | |
| I-II | | Reference | |  | |  | |  | |  | |
| III-IV | | 1.29(0.68-2.43) | |  | |  | |  | |  | |
| Diabetes | |  | | 0.64 | |  | |  | |  | |
| None | | Reference | |  | |  | |  | |  | |
| Yes | | 1.18 (0.59-2.36) | |  | |  | |  | |  | |
| Albumin (g/L) | |  | | 0.02 | |  | |  | | 0.01 | |
| ≤35 | | Reference | |  | |  | | Reference | |  | |
| ＞35 | | 0.43 (0.21-0.86) | |  | |  | | 0.38 (0.18-0.80） | |  | |
| CA199 (U/ml) | |  | | 0.87 | |  | |  | |  | |
| ≤143.7 | | Reference | |  | |  | |  | |  | |
| ＞143.7 | | 1.05 (0.56-1.99) | |  | |  | |  | |  | |
| PBD | |  | | 0.78 | |  | |  | |  | |
| None | | Reference | |  | |  | |  | |  | |
| Yes | | 1.1 (0.56-2.18) | |  | |  | |  | |  | |
| Surgery method | |  | | 0.6 | |  | |  | |  | |
| OPD | | Reference | |  | |  | |  | |  | |
| LPD | | 0.54 (0.06-5.35) | |  | |  | |  | |  | |
| Differentiation | |  | | 0.64 | |  | |  | |  | |
| Well/Moderate | | Reference | |  | |  | |  | |  | |
| Poor | | 1.18 (0.59-2.36) | |  | |  | |  | |  | |
| Tumor size | |  | | 0.2 | |  | |  | |  | |
| ≤3.0 | | Reference | |  | |  | |  | |  | |
| ＞3.0 | | 1.53 (0.80-2.91) | |  | |  | |  | |  | |
| Lymph metastasis | | | | <0.001 | |  | |  | | 0.02 | |
| None | | Reference | |  | |  | | Reference | |  | |
| Yes | | 3.70 (1.76-7.77) | |  | |  | | 3.53 (1.17-10.64) | |  | |
| Vascular invasion | | | | 0.87 | |  | |  | |  | |
| None | | Reference | |  | |  | |  | |  | |
| Yes | | 0.95 (0.50-1.79) | |  | |  | |  | |  | |
| TNM Stage | |  | | 0.01 | |  | |  | |  | |
| I | | Reference | |  | |  | | Reference | | 0.7 | |
| II-III | | 2.79 (1.32-5.88) | |  | |  | | 1.25 (0.41-3.82) | |  | |
| Chemotherapy | |  | | 0.25 | |  | |  | |  | |
| None | | Reference | |  | |  | |  | |  | |
| Yes | | 0.69 (0.36-1.31) | |  | |  | |  | |  | |
| Sarcopenia | |  | | 0.03 | |  | |  | | 0.07 | |
| None | | Reference | |  | |  | | Reference | |  | |
| Yes | | 2.08 (1.09-3.98) | |  | |  | | 1.89 (0.95-3.77) | |  | |
| NLR | | 1.06 (0.95-1.17) | | 0.29 | |  | |  | |  | |

Supplementary Table 3 Univariate and multivariate analysis of variables that predict overall complications in PC of Fujiwara criteria

| \| Variables \| Univariate regression \| P value \|  \| Multivariate regression \| P value \| \| --- \| --- \| --- \| --- \| --- \| --- \| \|  \| OR (95% CI ) \|  \|  \| OR (95% CI ) \|  \| \| Age (Years） \|  \| 0.12 \|  \|  \|  \| \| ≤65 \| Reference \|  \|  \|  \|  \| \| ＞65 \| 0.60 (0.31-1.15) \|  \|  \|  \|  \| \| Gender \|  \| 0.15 \|  \|  \|  \| \| Female \| Reference \|  \|  \|  \|  \| \| Male \| 1.61 (0.84-3.08) \|  \|  \|  \|  \| \| BMI (Kg/m2) \|  \| 0.56 \|  \|  \|  \| \| ≤24 \| Reference \|  \|  \|  \|  \| \| ＞24 \| 0.82 (0.41-1.61) \|  \|  \|  \|  \| \| ASA \|  \| 0.44 \|  \|  \|  \| \| I-II \| Reference \|  \|  \|  \|  \| \| III-IV \| 1.29(0.68-2.43) \|  \|  \|  \|  \| \| Diabetes \|  \| 0.64 \|  \|  \|  \| \| None \| Reference \|  \|  \|  \|  \| \| Yes \| 1.18 (0.59-2.36) \|  \|  \|  \|  \| \| Albumin (g/L) \|  \| 0.02 \|  \|  \| 0.007 \| \| ≤35 \| Reference \|  \|  \| Reference \|  \| \| ＞35 \| 0.43 (0.21-0.86) \|  \|  \| 0.36 (0.17-0.76） \|  \| \| CA199 (U/ml) \|  \| 0.87 \|  \|  \|  \| \| ≤143.7 \| Reference \|  \|  \|  \|  \| \| ＞143.7 \| 1.05 (0.56-1.99) \|  \|  \|  \|  \| \| PBD \|  \| 0.78 \|  \|  \|  \| \| None \| Reference \|  \|  \|  \|  \| \| Yes \| 1.1 (0.56-2.18) \|  \|  \|  \|  \| \| Surgery method \|  \|  \|  \|  \|  \| \| OPD \| Reference \| 0.6 \|  \|  \|  \| \| LPD \| 0.54 (0.06-5.35) \|  \|  \|  \|  \| \| Differentiation \|  \| 0.64 \|  \|  \|  \| \| Well/Moderate \| Reference \|  \|  \|  \|  \| \| Poor \| 1.18 (0.59-2.36) \|  \|  \|  \|  \| \| Tumor size \|  \| 0.01 \|  \|  \|  \| \| ≤3.0 \| Reference \|  \|  \|  \|  \| \| ＞3.0 \| 2.79 (1.32-5.88) \|  \|  \|  \|  \| \| Lymph metastasis \|  \| <0.001 \|  \|  \| 0.02 \| \| None \| Reference \|  \|  \| Reference \|  \| \| Yes \| 3.70 (1.76-7.77) \|  \|  \| 3.63 (1.25-10.57) \|  \| \| Vascular invasion \|  \| 0.87 \|  \|  \|  \| \| None \| Reference \|  \|  \|  \|  \| \| Yes \| 0.95 (0.50-1.79) \|  \|  \|  \|  \| \| TNM Stage \|  \| 0.01 \|  \|  \|  \| \| I \| Reference \|  \|  \| Reference \| 0.7 \| \| II-III \| 2.79 (1.32-5.88) \|  \|  \| 1.23 (0.42-3.67) \|  \| \| Chemotherapy \|  \| 0.25 \|  \|  \|  \| \| None \| Reference \|  \|  \|  \|  \| \| Yes \| 0.69 (0.36-1.31) \|  \|  \|  \|  \| \| Sarcopenia \|  \| 0.09 \|  \|  \|  \| \| None \| Reference \|  \|  \|  \|  \| \| Yes \| 2.25 (0.87-5.79) \|  \|  \|  \|  \| \| NLR \| 1.06 (0.95-1.17) \| 0.29 \|  \|  \|  \| |  |  |  |  |  |
| --- | --- | --- | --- | --- | --- | --- | --- | --- | --- | --- | --- | --- | --- | --- | --- | --- | --- | --- | --- | --- | --- | --- | --- | --- | --- | --- | --- | --- | --- | --- | --- | --- | --- | --- | --- | --- | --- | --- | --- | --- | --- | --- | --- | --- | --- | --- | --- | --- | --- | --- | --- | --- | --- | --- | --- | --- | --- | --- | --- | --- | --- | --- | --- | --- | --- | --- | --- | --- | --- | --- | --- | --- | --- | --- | --- | --- | --- | --- | --- | --- | --- | --- | --- | --- | --- | --- | --- | --- | --- | --- | --- | --- | --- | --- | --- | --- | --- | --- | --- | --- | --- | --- | --- | --- | --- | --- | --- | --- | --- | --- | --- | --- | --- | --- | --- | --- | --- | --- | --- | --- | --- | --- | --- | --- | --- | --- | --- | --- | --- | --- | --- | --- | --- | --- | --- | --- | --- | --- | --- | --- | --- | --- | --- | --- | --- | --- | --- | --- | --- | --- | --- | --- | --- | --- | --- | --- | --- | --- | --- | --- | --- | --- | --- | --- | --- | --- | --- | --- | --- | --- | --- | --- | --- | --- | --- | --- | --- | --- | --- | --- | --- | --- | --- | --- | --- | --- | --- | --- | --- | --- | --- | --- | --- | --- | --- | --- | --- | --- | --- | --- | --- | --- | --- | --- | --- | --- | --- | --- | --- | --- | --- | --- | --- | --- | --- | --- | --- | --- | --- | --- | --- | --- | --- | --- | --- | --- | --- | --- | --- | --- | --- | --- | --- | --- | --- | --- | --- | --- | --- | --- | --- | --- | --- | --- | --- | --- | --- | --- | --- | --- | --- | --- | --- | --- | --- | --- | --- | --- | --- | --- | --- | --- | --- | --- | --- | --- | --- | --- | --- | --- | --- | --- | --- | --- | --- | --- | --- | --- | --- | --- | --- | --- | --- | --- | --- | --- | --- | --- | --- | --- | --- | --- | --- | --- | --- | --- | --- | --- | --- | --- | --- | --- | --- | --- | --- | --- | --- | --- | --- | --- | --- |

Supplementary Table 4 Univariate and multivariate Cox regression analysis of variables related to OS in PC of Asian pancreatic cancer population sarcopenia criteria

| Variables | | Univariate Cox regression | | P value | |  | Multivariate Cox regression | | P value |
| --- | --- | --- | --- | --- | --- | --- | --- | --- | --- |
|  | | HR (95% CI ) | |  | |  | HR (95% CI ) | |  |
| Age (years) |  | | 0.61 | |  | | |  |  |
| ≤65 | Reference | |  | |  | | |  |  |
| ＞65 | 1.09 (0.78-1.53) | |  | |  | | |  |  |
| Gender |  | | 0.74 | |  | | |  |  |
| Female | Reference | |  | |  | | |  |  |
| Male | 1.06 (0.76-1.48) | |  | |  | | |  |  |
| BMI (Kg/m2) |  | | 0.19 | |  | | |  |  |
| ≤24 | Reference | |  | |  | | |  |  |
| ＞24 | 0.79 (0.55-1.13) | |  | |  | | |  |  |
| ASA |  | | 0.58 | |  | | |  |  |
| I-II | Reference | |  | |  | | |  |  |
| III-IV | 0.91 (0.65-1.27) | |  | |  | | |  |  |
| Diabetes |  | | 0.28 | |  | | |  |  |
| None | Reference | |  | |  | | |  |  |
| Yes | 0.81 (0.56-1.18) | |  | |  | | |  |  |
| Albumin |  | | 0.76 | |  | | |  |  |
| ≤35 | Reference | |  | |  | | |  |  |
| ＞35 | 1.06 (0.73-1.53) | |  | |  | | |  |  |
| CA199 |  | | <0.001 | |  | | |  | 0.035 |
| ≤143.7 | Reference | |  | |  | | | Reference |  |
| ＞143.7 | 1.78 (1.27-2.50) | |  | |  | | | 1.46 (1.03-2.10) |  |
| PBD |  | | 0.37 | |  | | |  |  |
| None | Reference | |  | |  | | |  |  |
| Yes | 0.85 (0.59-1.22) | |  | |  | | |  |  |
| Differentiation |  | | ＜0.001 | |  | | |  | ＜0.001 |
| Well/Moderate | Reference | |  | |  | | | Reference |  |
| Poor | 2.53 (1.74-3.66) | |  | |  | | | 2.21 (1.49-3.30) |  |
| Tumor size |  | | 0.001 | |  | | |  | 0.013 |
| ≤3.0 | Reference | |  | |  | | | Reference |  |
| ＞3.0 | 1.77 (1.26-2.48) | |  | |  | | | 1.61 (1.10-2.40) |  |
| Lymph metastasis |  | | 0.01 | |  | | |  | 0.466 |
| None | Reference | |  | |  | | | Reference |  |
| Yes | 1.60 (1.12-2.28) | |  | |  | | | 0.83 (0.50-1.40) |  |
| Vascular invasion |  | | 0.028 | |  | | |  | 0.639 |
| None | Reference | |  | |  | | | Reference |  |
| Yes | 1.46 (1.04 -2.05) | |  | |  | | | 1.09 (0.76-1.60) |  |
| TNM Stage |  | | ＜0.001 | |  | | |  | 0.009 |
| I | Reference | |  | |  | | | Reference |  |
| II-III | 2.51 (1.69-3.72) | |  | |  | | | 2.12 (1.22-4.00) |  |
| Chemotherapy |  | |  | |  | | |  |  |
| Gemcitabine | Reference | |  | |  | | |  |  |
| FOLFIRINOX+unknown | 1.23 (0.75-2.00) | | 0.41 | |  | | |  |  |
| None | 1.41 (0.97-2.04) | | 0.07 | |  | | |  |  |
| Sarcopenia |  | | ＜0.001 | |  | | |  | ＜0.001 |
| None | Reference | |  | |  | | | Reference |  |
| Yes | 2.52 (1.78-3.55) | |  | |  | | | 2.59 (1.80-3.70) |  |
| NLR | 0.97 (0.91-1.02) | | 0.25 | |  | | |  |  |

Supplementary Table 5 Univariate and multivariate Cox regression analysis of variables related to RFS in PC of Asian pancreatic cancer population sarcopenia criteria

| Variables | Univariate Cox regression | | | P value | |  | | Multivariate Cox regression | | | P value |
| --- | --- | --- | --- | --- | --- | --- | --- | --- | --- | --- | --- |
|  | HR (95% CI ) | | |  | |  | | HR (95% CI ) | | |  |
| Age (Years) | | | 0.42 | |  | |  | |  |  |  |
| ≤65 | | Reference |  | |  | |  | |  |  |  |
| ＞65 | | 0.85 (0.58-1.25) |  | |  | |  | |  |  |  |
| Gender | |  | 0.68 | |  | |  | |  |  |  |
| Female | | Reference |  | |  | |  | |  |  |  |
| Male | | 1.08 (0.74-1.58) |  | |  | |  | |  |  |  |
| BMI (Kg/m2) | | | 0.16 | |  | |  | |  |  |  |
| ≤24 | | Reference |  | |  | |  | |  |  |  |
| ＞24 | | 0.75 (0.50-1.12) |  | |  | |  | |  |  |  |
| ASA | |  | 0.24 | |  | |  | |  |  |  |
| I-II | | Reference |  | |  | |  | |  |  |  |
| III-IV | | 0.80 (0.54-1.16) |  | |  | |  | |  |  |  |
| Diabetes | |  | 0.22 | |  | |  | |  |  |  |
| None | | Reference |  | |  | |  | |  |  |  |
| Yes | | 0.77 (0.50-1.18) |  | |  | |  | |  |  |  |
| Albumin (g/L) | |  | 0.16 | |  | |  | |  |  |  |
| ≤35 | | Reference |  | |  | |  | |  |  |  |
| ＞35 | | 1.37 (0.88-2.12) |  | |  | |  | |  |  |  |
| CA199 (U/ml) | |  | 0.07 | |  | |  | |  |  |  |
| ≤143.7 | | Reference |  | |  | |  | |  |  |  |
| ＞143.7 | | 1.43 (0.98-2.08) |  | |  | |  | |  |  |  |
| PBD | |  | 0.32 | |  | |  | |  |  |  |
| None | | Reference |  | |  | |  | |  |  |  |
| Yes | | 0.81 (0.53-1.23) |  | |  | |  | |  |  |  |
| Differentiation | | | ＜0.001 | |  | |  | | ＜0.001 |  |  |
| Well/Moderate | | Reference |  | |  | | Reference | |  |  |  |
| Poor | | 2.74 (1.81-4.13) |  | |  | | 2.40 (1.59-3.80) | |  |  |  |
| Tumor size (cm) | | | 0.002 | |  | |  | | 0.053 |  |  |
| ≤3.0 | | Reference |  | |  | | Reference | |  |  |  |
| ＞3.0 | | 1.82 (1.24-2.68) |  | |  | | 1.50 (0.99-2.30) | |  |  |  |
| Lymph metastasis | | | ＜0.001 | |  | |  | | 0.034 |  |  |
| None | | Reference |  | |  | | Reference | |  |  |  |
| Yes | | 3.46 (2.16-5.56) |  | |  | | 2.00 (1.05-3.90) | |  |  |  |
| Vascular invasion | | | 0.13 | |  | |  | |  |  |  |
| None | | Reference |  | |  | |  | |  |  |  |
| Yes | | 1.35 (0.92 -1.97) |  | |  | |  | |  |  |  |
| TNM Stage | | | ＜0.001 | |  | |  | | 0.033 |  |  |
| I | | Reference |  | |  | | Reference | |  |  |  |
| II-III | | 3.96 (2.37-6.60) |  | |  | | 2.20 (1.07-4.50) | |  |  |  |
| Chemotherapy | | |  | |  | |  | |  |  |  |
| Gemcitabine | | Reference |  | |  | |  | |  |  |  |
| FOLFIRINOX + Unknown | | 1.10 (0.63-1.91) | 0.74 | |  | |  | |  |  |  |
| None | | 1.18 (0.78-1.78) | 0.44 | |  | |  | |  |  |  |
| Sarcopenia | | | ＜0.001 | |  | |  | | ＜0.001 |  |  |
| None | | Reference |  | |  | | Reference | |  |  |  |
| Yes | | 2.14 (1.46-3.14) |  | |  | | 2.00 (1.36-3.00) | |  |  |  |
| NLR | | 0.97 (0.91-1.04) | 0.34 | |  | |  | |  |  |  |

Supplementary Table 6 Univariate and multivariate Cox regression analysis of variables related to OS in PC of Fujiwara criteria

| Variables | | Univariate Cox regression | | P value | |  | Multivariate Cox regression | | P value |
| --- | --- | --- | --- | --- | --- | --- | --- | --- | --- |
|  | | HR (95% CI ) | |  | |  | HR (95% CI ) | |  |
| Age (Years) |  | | 0.61 | |  | | |  |  |
| ≤65 | Reference | |  | |  | | |  |  |
| ＞65 | 1.09 (0.78-1.53) | |  | |  | | |  |  |
| Gender |  | | 0.74 | |  | | |  |  |
| Female | Reference | |  | |  | | |  |  |
| Male | 1.06 (0.76-1.48) | |  | |  | | |  |  |
| BMI (Kg/m2) |  | | 0.19 | |  | | |  |  |
| ≤24 | Reference | |  | |  | | |  |  |
| ＞24 | 0.79 (0.55-1.13) | |  | |  | | |  |  |
| ASA |  | | 0.58 | |  | | |  |  |
| I-II | Reference | |  | |  | | |  |  |
| III-IV | 0.91 (0.65-1.27) | |  | |  | | |  |  |
| Diabetes |  | | 0.28 | |  | | |  |  |
| None | Reference | |  | |  | | |  |  |
| Yes | 0.81 (0.56-1.18) | |  | |  | | |  |  |
| Albumin |  | | 0.76 | |  | | |  |  |
| ≤35 | Reference | |  | |  | | |  |  |
| ＞35 | 1.06 (0.73-1.53) | |  | |  | | |  |  |
| CA199 |  | | <0.001 | |  | | |  | 0.026 |
| ≤143.7 | Reference | |  | |  | | | Reference |  |
| ＞143.7 | 1.78 (1.27-2.50) | |  | |  | | | 1.49 (1.05-2.10) |  |
| PBD |  | | 0.37 | |  | | |  |  |
| None | Reference | |  | |  | | |  |  |
| Yes | 0.85 (0.59-1.22) | |  | |  | | |  |  |
| Differentiation |  | | ＜0.001 | |  | | |  | ＜0.001 |
| Well/Moderate | Reference | |  | |  | | | Reference |  |
| Poor | 2.53 (1.74-3.66) | |  | |  | | | 2.32 (1.57-3.40) |  |
| Tumor size |  | | 0.001 | |  | | |  | 0.042 |
| ≤3.0 | Reference | |  | |  | | | Reference |  |
| ＞3.0 | 1.77 (1.26-2.48) | |  | |  | | | 1.47 (1.01-2.10) |  |
| Lymph metastasis |  | | 0.01 | |  | | |  | 0.822 |
| None | Reference | |  | |  | | | Reference |  |
| Yes | 1.60 (1.12-2.28) | |  | |  | | | 0.95 (0.58-1.50) |  |
| Vascular invasion |  | | 0.028 | |  | | |  | 0.705 |
| None | Reference | |  | |  | | | Reference |  |
| Yes | 1.46 (1.04 -2.05) | |  | |  | | | 1.07 (0.75-1.50) |  |
| TNM Stage |  | | ＜0.001 | |  | | |  | 0.007 |
| I | Reference | |  | |  | | | Reference |  |
| II-III | 2.51 (1.69-3.72) | |  | |  | | | 2.17 (1.24-3.80) |  |
| Chemotherapy |  | |  | |  | | |  |  |
| Gemcitabine | Reference | |  | |  | | |  |  |
| FOLFIRINOX+Unknown | 1.23 (0.75-2.00) | | 0.41 | |  | | |  |  |
| None | 1.41 (0.97-2.04) | | 0.07 | |  | | |  |  |
| Sarcopenia |  | | 0.003 | |  | | |  |  |
| None | Reference | |  | |  | | | Reference | 0.025 |
| Yes | 2.10 (1.30-3.40) | |  | |  | | | 1.81 (1.08-3.10) |  |
| NLR | 0.97 (0.91-1.02) | | 0.25 | |  | | |  |  |

Supplementary Table 7 Univariate and multivariate Cox regression analysis of variables related to RFS in PC of Fujiwara criteria

| Variables | Univariate Cox regression | | | P value | | |  | Multivariate Cox regression | | | P value |
| --- | --- | --- | --- | --- | --- | --- | --- | --- | --- | --- | --- |
|  | HR (95% CI ) | | |  | | |  | HR (95% CI ) | | |  |
| Age (Years) | | | 0.42 | |  |  | | |  |  |  |
| ≤65 | | Reference |  | |  |  | | |  |  |  |
| ＞65 | | 0.85 (0.58-1.25) |  | |  |  | | |  |  |  |
| Gender | |  | 0.68 | |  |  | | |  |  |  |
| Female | | Reference |  | |  |  | | |  |  |  |
| Male | | 1.08 (0.74-1.58) |  | |  |  | | |  |  |  |
| BMI (Kg/m2) | | | 0.16 | |  |  | | |  |  |  |
| ≤24 | | Reference |  | |  |  | | |  |  |  |
| ＞24 | | 0.75 (0.50-1.12) |  | |  |  | | |  |  |  |
| ASA | |  | 0.24 | |  |  | | |  |  |  |
| I-II | | Reference |  | |  |  | | |  |  |  |
| III-IV | | 0.80 (0.54-1.16) |  | |  |  | | |  |  |  |
| Diabetes | |  | 0.22 | |  |  | | |  |  |  |
| None | | Reference |  | |  |  | | |  |  |  |
| Yes | | 0.77 (0.50-1.18) |  | |  |  | | |  |  |  |
| Albumin (g/L) | |  | 0.16 | |  |  | | |  |  |  |
| ≤35 | | Reference |  | |  |  | | |  |  |  |
| ＞35 | | 1.37 (0.88-2.12) |  | |  |  | | |  |  |  |
| CA199 (U/ml) | |  | 0.07 | |  |  | | |  |  |  |
| ≤143.7 | | Reference |  | |  |  | | |  |  |  |
| ＞143.7 | | 1.43 (0.98-2.08) |  | |  |  | | |  |  |  |
| PBD | |  | 0.32 | |  |  | | |  |  |  |
| None | | Reference |  | |  |  | | |  |  |  |
| Yes | | 0.81 (0.53-1.23) |  | |  |  | | |  |  |  |
| Differentiation | | | ＜0.001 | |  |  | | | ＜0.001 |  |  |
| Well/Moderate | | Reference |  | |  | Reference | | |  |  |  |
| Poor | | 2.74 (1.81-4.13) |  | |  | 2.50 (1.65-3.90) | | |  |  |  |
| Tumor size (cm) | | | 0.002 | |  |  | | | 0.075 |  |  |
| ≤3.0 | | Reference |  | |  | Reference | | |  |  |  |
| ＞3.0 | | 1.82 (1.24-2.68) |  | |  | 1.40 (0.96-2.20) | | |  |  |  |
| Lymph metastasis | | | ＜0.001 | |  |  | | | 0.014 |  |  |
| None | | Reference |  | |  | Reference | | |  |  |  |
| Yes | | 3.46 (2.16-5.56) |  | |  | 2.20 (1.17-4.00) | | |  |  |  |
| Vascular invasion | | | 0.13 | |  |  | | |  |  |  |
| None | | Reference |  | |  |  | | |  |  |  |
| Yes | | 1.35 (0.92 -1.97) |  | |  |  | | |  |  |  |
| TNM Stage | | | ＜0.001 | |  |  | | | 0.022 |  |  |
| I | | Reference |  | |  | Reference | | |  |  |  |
| II-III | | 3.96 (2.37-6.60) |  | |  | 2.20 (1.12-4.40) | | |  |  |  |
| Chemotherapy | | |  | |  |  | | |  |  |  |
| Gemcitabine | | Reference |  | |  |  | | |  |  |  |
| FOLFIRINOX + Unknown | | 1.10 (0.63-1.91) | 0.74 | |  |  | | |  |  |  |
| None | | 1.18 (0.78-1.78) | 0.44 | |  |  | | |  |  |  |
| Sarcopenia | | | 0.042 | |  |  | | |  |  |  |
| None | | Reference |  | |  | Reference | | | 0.10 |  |  |
| Yes | | 1.80 (1.02-3.18) |  | |  | 1.60 (0.90-3.00) | | |  |  |  |
| NLR | | 0.97 (0.91-1.04) | 0.34 | |  |  | | |  |  |  |
